# Neonatal-onset multiple acyl-CoA dehydrogenase deficiency (MADD) in the *ETFDH* gene: A case report and a literature review

**DOI:** 10.1097/MD.0000000000021944

**Published:** 2020-09-11

**Authors:** Meijuan Ding, Ruihua Liu, Li Qiubo, Yanke Zhang, Qingxia Kong

**Affiliations:** a Jining Medical University; b Department of Pediatric, Affiliated Hospital of Jining Medical University; c Department of Neurology, Affiliated Hospital of Jining Medical University, Jining, Shandong, P.R. China.

**Keywords:** ETFDH, glutaric aciduria type II, MADD, review

## Abstract

**Rationale:**

Multiple acyl-CoA dehydrogenase deficiency (MADD) is a rare inborn error of metabolism affecting fatty acid, amino acid, and choline metabolism. The clinical manifestation of MADD is heterogeneous, from severe neonatal forms to mild late-onset forms.

**Patient concerns:**

Here, we report a patient who presented with severe hypoglycemia and exercise intolerance suggestive of MADD. Serum tandem mass spectrometry analysis indicated elevated levels of various acyl carnitines at 25 days of age. Exome sequencing of the proband revealed compound heterozygous mutations, c. 413T>G (p.Leu138Arg) and c.1667C > G (p.Pro556Arg), in the ETFDH gene as the probable causative mutations.

**Diagnoses:**

Based on the patient's clinical presentation and test results, the patient was diagnosed with MADD.

**Interventions:**

A high-calorie and reduced-fat diet was given together with oral supplements of L-carnitine (150 mg/day).

**Outcomes:**

He passed away at the age of 4 months because of severe respiratory distress accompanied by muscle weakness.

**Lessons:**

He passed away at the age of 4 months because of severe respiratory distress accompanied by muscle weakness. Clinicians should consider MADD in the differential diagnosis when patients present with muscle weakness and biochemical abnormalities. Gene testing plays a critical role in confirming the diagnosis of MADD and may not only prevent the need for invasive testing but also allow for timely initiation of treatment.

## 1 Introduction

Multiple Acyl-CoA dehydrogenase deficiency (MADD), also known as glutaric aciduria type II (GA II), is a rare autosomal recessive disorder of fatty acid, amino acid‘, and choline metabolism caused by a defect in the alpha or beta subunit of the mitochondrial electron transfer flavoprotein (ETFA, ETFB) protein or the electron transfer flavoprotein dehydrogenase (ETFDH) protein.^[1]^ This is a clinically heterogeneous disease that has been divided into three clinical forms: a neonatal-onset form with congenital anomalies (type I), a neonatal-onset form without congenital anomalies (type II), and a late-onset form (type III).^[2]^ The condition is clinically heterogeneous ranging from a severe, neonatal form, presenting with hypoketotic hypoglycemia, metabolic acidosis, cardiomyopathy, and hepatomegaly to a later-onset form characterized by proximal myopathy.^[3]^ Types I and II are severe, typically fatal, and characterized by nonketotic hypoglycemia, metabolic acidosis, and accumulation and excretion of metabolites, while Type III is milder, more variable, and characterized by recurrent episodes of hypoglycemia, metabolic acidosis, vomiting, and muscle weakness during catabolic stress.^[4]^ The biochemical characterization of MADD includes organic acid and acylcarnitine profiling, which will reveal increased levels of aliphatic mono- and dicarboxylic acids, acylglycine conjugates as well as increases in C4-C18 acylcarnitines in the blood.

Here, we report a case of early onset MADD characterized by hypoglycemia and progressive muscle weakness in which a novel compound heterozygous mutation within the *ETFDH* gene was identified.

## 2 Case

A male neonate was born at term as the first child to nonconsanguineous parents after a normal pregnancy and an uneventful delivery, with a weight of 3300 g (50th–90th percentile). His Apgar score was 8. His blood glucose varied from 0.7 to 2.8 mmol/L (reference interval [RI]: 4.2–6.3 mmol/L) during the first 24 h of his life and has denormalized since. The patient was transferred to the neonatal intensive care unit of the local hospital on the second day of life following onset of hypotonia, feeding problems, frequent periods of vomiting, hyperammonemia, and hypoglycemia. Blood acylcarnitine profiles showed a combined elevation of long and medium chain acylcarnitines, such as butyryl carnitine (C4), octanoylcarnitine (C8), decanoylcarnitine (C10), dodecanoylcarnitine (C12), and myristoylcarnitine (C14), supporting a diagnosis of MADD. Laboratory Investigations: ALT activity in plasma was elevated up to 78.2 U/L (normal < 50 U/L), AST was 292 U/L (normal < 40), lactate was 6.5 mmol/L (normal < 2.1), plasma ammonia was 59.6 (normal < 33 umol/L), and creatine kinase isoenzymes were 13.94 ng/mL (normal < 6.3).

**Figure 1:**
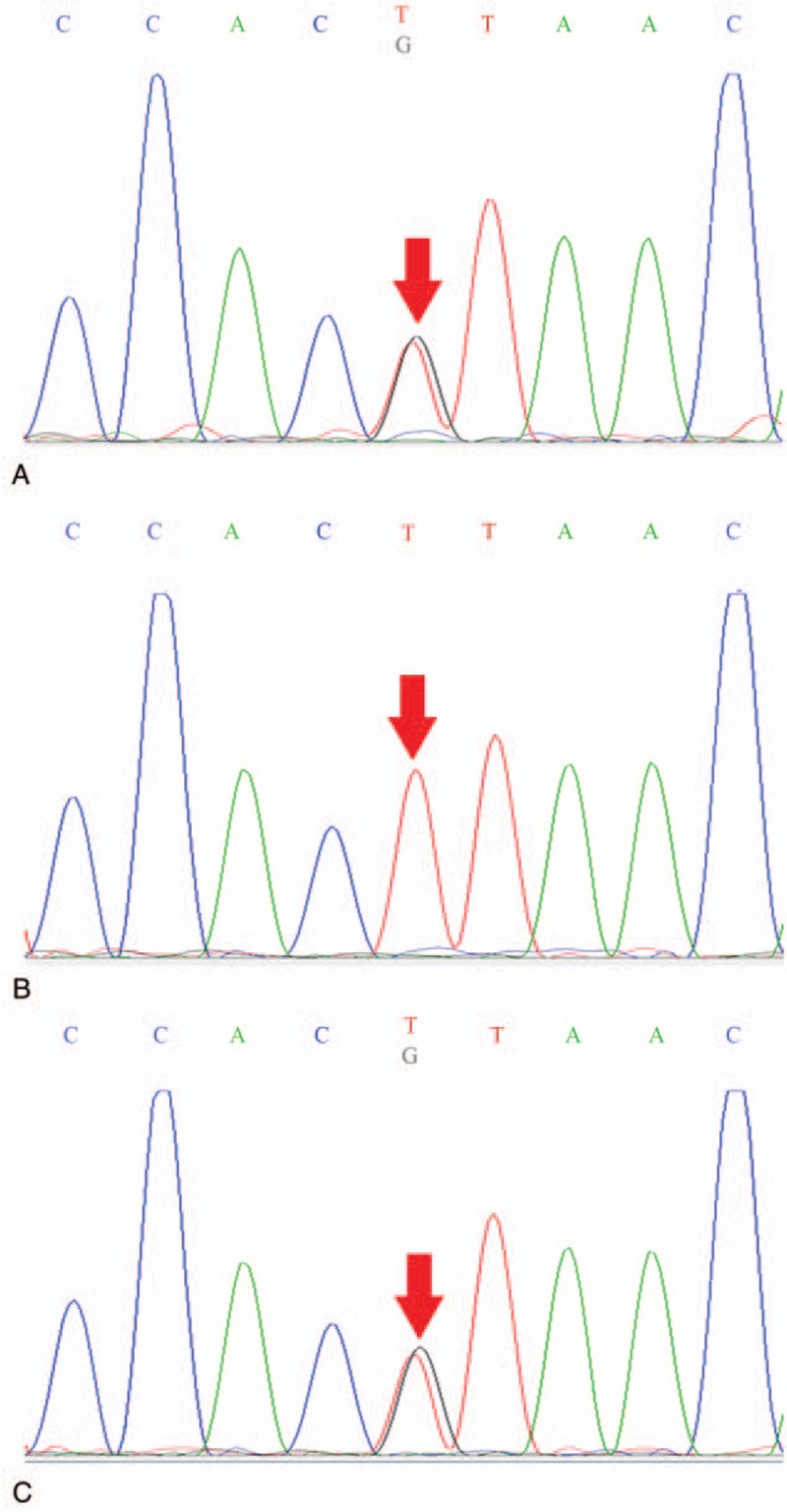
(A) The child's *ETFDH* gene has a missense mutation. 413T > G (coding region nucleotide number 413, from T to G) heterozygous nucleotide variation led to amino acid number 138 changing from Leu to Arg (p. Leu138Arg). (B) His father's *ETFDH* gene did not have the mutation (arrow). (C) His mother's *ETFDH* gene has the same mutation (arrow).

**Figure 2:**
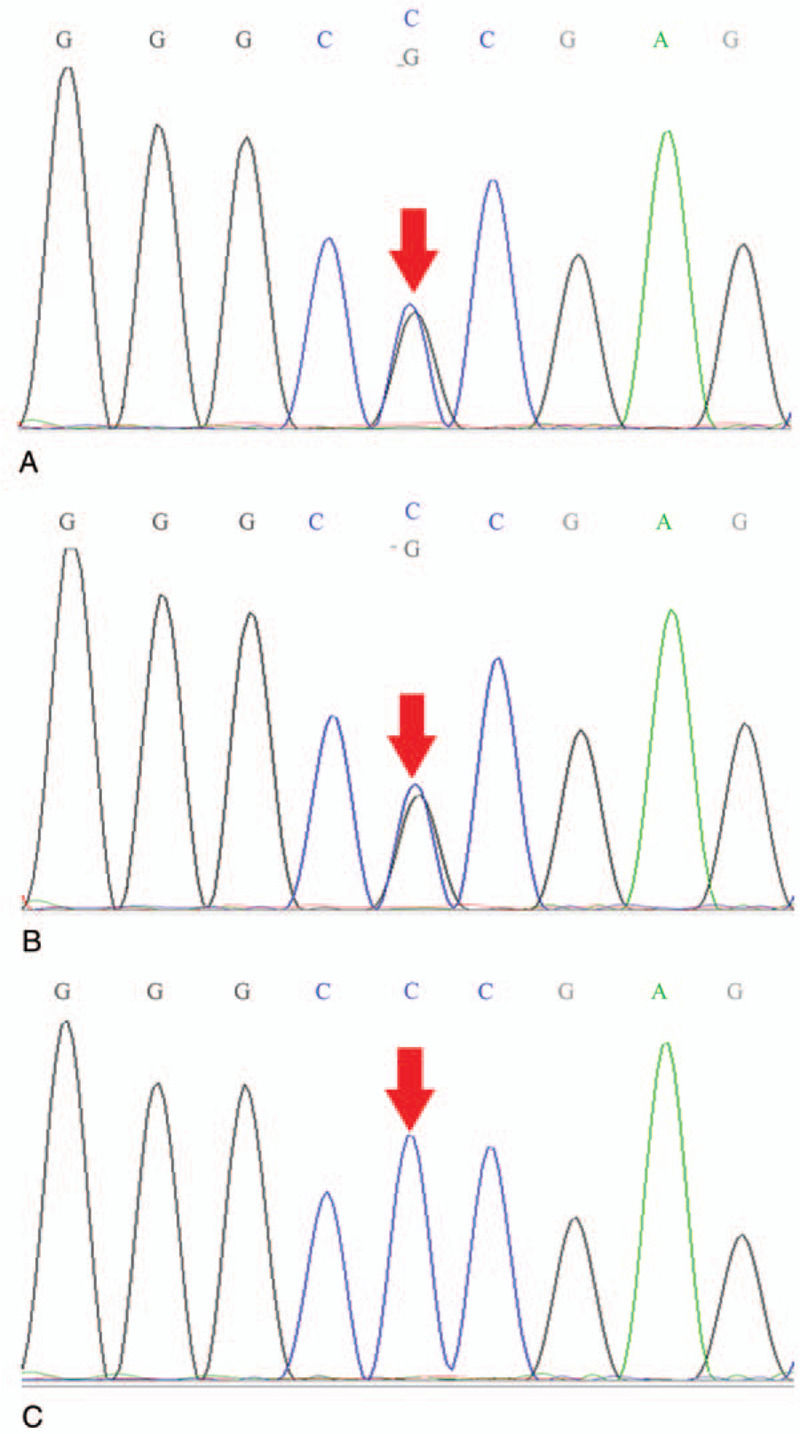
(A) The child's *ETFDH* gene has a missense mutation. 1667C > G (coding region nucleotide number 1667, from C to G) heterozygous nucleotide variation led to amino acid number 556 changing from Pro to Arg (p. Pra556Arg). (B) His father's *ETFDH* gene has the same mutation (arrow). (C) His mother's *ETFDH* gene is normal (arrow).

To further confirm the diagnosis, we sequenced the subtotal exome genes of the patient and his parents and identified mutations in the *ETFDH* gene in the complex of c. 413T> G (nucleotide 413 in the coding region changed thymine to guanine) and c. 1667C> G (nucleotide 1667 of coding region changed cytosine to guanine) in exon 4 (Figs. 1 and 2). These mutations resulted in the transformation of amino acid 556 from proline to arginine (p.Pro556 Arg) and amino acid 138 from leucine to arginine (p.Leu138Arg). Both were missense mutations. The above loci were inherited from the parents, each of whom carried only one heterozygous variant. Molecular diagnosis of the mother showed the heterozygous mutation in exon 4 c. 413T > G (p.Leu138Arg), and that of the father showed the heterozygous mutation in exon 12 c.1667C > G (p.Pro556Arg).

**Figure 3:**
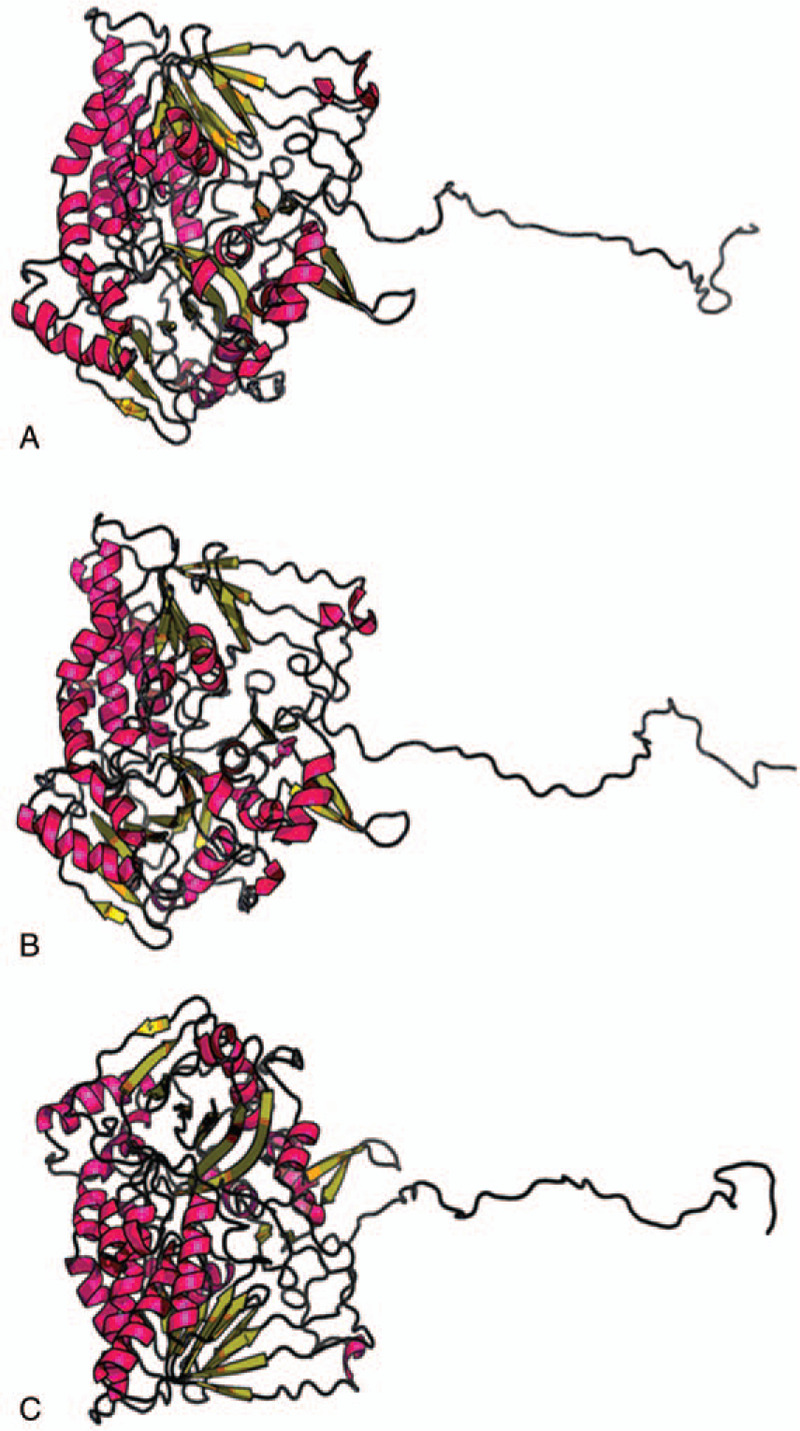
The tertiary structure of the two is significantly different. (A) shows the tertiary structure of the normal ETFDH protein. (B) shows the tertiary structure of the amino acid number 138 changing from Leu to Arg (p. Leu138Arg). (C) shows the tertiary structure of the amino acid number 556 changing from Pro to Arg (p. Pra556Arg).

To further investigate the structure, function and interaction of potential changes in this gene, we used the method of D’Angelo R using CLC Genomics workbench 8.0.1 (www.clcbio.com), PSIPRED (http://bioinf.cs.ucl.ac.uk/psipred/), RaptorX (http://raptorx.uchicago.edu) and Chimera (http://www.cgl.ucsf.edu/chimera/) to predict the primary, secondary, and tertiary structure of the ETFDH protein separately. According to the three-dimensional view, it was predicted that the gene mutation caused changes in the spatial structure, which led to a change in protein translation and modification (Fig. 3).

According to the clinical manifestations, the biochemical examination and the genetic testing of the patient, MADD was diagnosed. A high-calorie and reduced-fat diet was started together with oral supplements of L-carnitine (150 mg/day). After 1 week, the patient's clinical symptoms improved dramatically. His muscle weakness disappeared. However, after discharge, he repeatedly presented with episodic vomiting and hypoglycemia. The patient was found to have low muscle tension and motor function 2 months after birth. Rehabilitation therapy was given twice, and the symptoms improved. On day 115 after birth, poor feeding occurred again, accompanied by shortness of breath and vomiting. When he was 4 months old, he came to our hospital for emergency treatment because of severe respiratory distress accompanied by muscle weakness. Unfortunately, his condition continued to deteriorate and he passed away at the age of 4 months.

## 3 Discussion

In this study, we report two novel compound heterozygous mutations in the ETFDH gene in one patient with MADD from both families. The diagnosis of MADD was primarily based on biochemical data (increased levels of acylcarnitines), and confirmed by ETFDH mutation analysis.^[5]^ Although MADD is a treatable disease, it is rare and its diagnosis is difficult due to high clinical heterogeneity. In the emergency department, MADD patients often present with fluctuating muscle weakness, vomiting, hypoglycemia, metabolic acidosis, encephalopathy, and hepatopathy.^[6–8]^ However, many muscle diseases (such as inflammatory myopathy, metabolic myopathy, and progressive muscular dystrophy) are also associated with muscle weakness. Thus, MADD may be misdiagnosed as a different type of lipid storage myopathy, a glycogen storage disease, progressive muscular dystrophy, or other muscle disease,^[9]^ or gastrointestinal diseases.

According to a report, in most parts of the world and in the absence of consanguinity, MADD II is a rare neonatal disease that can present with or without congenital anomalies.^[10]^ We summarized the 4 cases of MADD II, as shown in the table below (Table 1). The clinical manifestations of the patients were serious, and the disease can manifest at a later stage as intermittent episodes of vomiting, acidosis, and hypoglycemia. It is easily and promptly diagnosed through a MS/MS study or genetic testing, which should be used as a screening tool for this otherwise lethal disorder. Treatment with riboflavin and L-carnitine were tried in all patients. In the four patients, only one survived. Other patients died shortly after the onset of the disease. Our patient did not survive the treatment. Therefore, timely and early regular treatment may be able to save patients, although the chances are very low. Treatment with special diet, riboflavin, and carnitine, especially the IV form, should be tried in all patients. At the same time, patient C showed us that the family of a patient can subsequently have a normal male child delivered after a prenatal diagnosis performed using chorionic villus sampling, which can provide new hope for the lives of patients’ families.

**Table 1:**
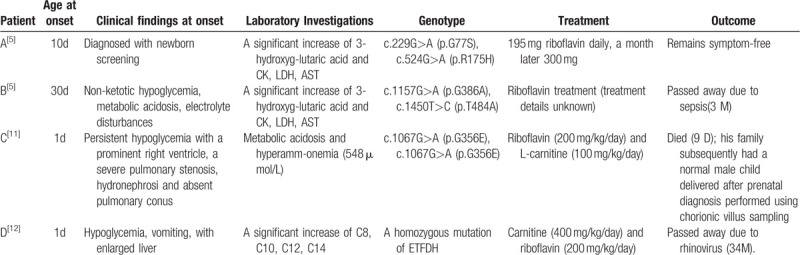
Overview of the four MADD patients.

Multiple acyl-CoA dehydrogenase deficiency (MADD), or glutaric aciduria type 2, affects metabolism of fatty and amino acids. The most commonly affected protein is electron transfer flavoprotein-ubiquinone oxidoreductase (ETFDH), encoded by ETFDH.^[13–15]^ The other known affected genes, ETFA and ETFB, encode the α and β subunits of the electron transfer flavoprotein, respectively.^[2,16]^ A mutation in any of these genes results in deficient electron transfer to the mitochondrial respiratory chain, thus reducing the energy production of the body. We identified the mutation of ETFDH gene in the complex of c. 413T> G and c. 1667C> G in exon 4. SIFT, Polyphen2, and MutationTaster were used for in silico analysis of candidate variants. Deleterious variants were confirmed by subtotal exon sequencing. No mutation was identified in *ETFA* or *ETFB* gene. Reports indicate that mutations are likely to cause compromised stability of the mutant proteins and further demonstrated that the cause of disease occurrence.^[5]^

Hot spots for mutations have been identified in Asian populations, with c.250G > A (p.A84T) the most common mutation in southern China and c.770A > G (p.G362R) and c.1227A > C (p.L409F) the most common mutations in northern China.^[14,15,17]^ Indeed, ETFDH mutations may be ethno-specific. In the MADD patients from northern China in this study, we identified two new mutations, one of which was the previously reported mutation in ETFDH. The new mutation is c.1667C > G (p.Pro556Arg) in exon 12, which expands the spectrum of mutations found in patients with MADD.

Currently, gene testing plays an important role in MADD diagnosis, and may prevent more invasive diagnostic testing, such as muscle biopsy. Given the current knowledge of the genetic etiology underlying MADD, we suggest a stepwise approach for patients suspected of having MADD, involving targeted sequencing of ETFA, ETFB, and ETFDH followed by WES if targeted sequencing is negative.^[5]^ MADD are multisystem genetic diseases characterized by various clinical manifestations with different degrees of severity. It is usually difficult to diagnose in an emergency department. The most common clinical phenotype is the type III (RR-MADD), often associated with *ETFDH* gene mutations. Although *ETFDH* gene mutations are present in this patient, they were not sensitive to riboflavin therapy, which may have missed the period of medication due to late administration. Therefore, timely treatment is essential for patients.^[18]^ The table of retrospective studies shows that for early onset MADD, timely diagnosis and adequate and correct treatment can prolong the life span of patients or provide a normal life. According to the reports, the family of this patient could subsequently have a normal child delivered after prenatal diagnosis performed using chorionic villus sampling, thus improving the quality of life of the family.

## Acknowledgments

We are indebted to Affiliated Hospital of Jining Medical College for assistance with data collection, to Teachers of Epilepsy Consultation Center who give us help.

## Author contributions

QK and QL contributed to the study design; MD and RL collected the clinical data and wrote the paper, YK analysed the data.
